# The effect of conditional EFNB1 deletion in the T cell compartment on T cell development and function

**DOI:** 10.1186/1471-2172-12-68

**Published:** 2011-12-19

**Authors:** Wei Jin, Shijie Qi, Hongyu Luo

**Affiliations:** 1 From the Laboratory of Immunology Centre de recherche de Centre hospitalier de l'Université de Montréal (CRCHUM), Notre-Dame Hospital, Montreal, Quebec, Canada

## Abstract

**Background:**

Eph kinases are the largest family of cell surface receptor tyrosine kinases. The ligands of Ephs, ephrins (EFNs), are also cell surface molecules. Ephs interact with EFNs transmitting signals in both directions, i.e., from Ephs to EFNs and from EFNs to Ephs. EFNB1 is known to be able to co-stimulate T cells *in vitro *and to modulate thymocyte development in a model of foetal thymus organ culture. To further understand the role of EFNB1 in T cell immunity, we generated T-cell-specific EFNB1 gene knockout mice to assess T cell development and function in these mice.

**Results:**

The mice were of normal size and cellularity in the thymus and spleen and had normal T cell subpopulations in these organs. The bone marrow progenitors from KO mice and WT control mice repopulated host spleen T cell pool to similar extents. The activation and proliferation of KO T cells was comparable to that of control mice. Naïve KO CD4 cells showed an ability to differentiate into Th1, Th2, Th17 and Treg cells similar to control CD4 cells.

**Conclusions:**

Our results suggest that the function of EFNB1 in the T cell compartment could be compensated by other members of the EFN family, and that such redundancy safeguards the pivotal roles of EFNB1 in T cell development and function.

## Background

Eph kinases are the largest family of cell surface receptor tyrosine kinases and can be divided into A and B subfamilies according to their sequence homology. The ligands of Ephs, ephrins (EFNs), are also cell surface molecules. EFNs are divided into A and B subfamilies. EFNAs are GPI-anchored cell surface proteins and EFNBs are transmembrane cell surface proteins. Ephs and EFNs interact promiscuously. EphA members mainly interact with EFNA members, while EphB members mainly interact with EFNB members. When Ephs and EFNs interact, signals are transmitted in both directions, i.e., from Ephs to EFNs and from EFNs to Ephs. Such bi-directional signalling is called forward and reversed signalling respectively [1].

EFNB1 is involved in the development and function of the central nervous system [2]. It is also essential in bone maintenance and remodelling [3]. EFNB1 expression in platelets contributes to the clotting process [4]. Its expression on kidney epithelial cells (podocytes) likely plays a role in glomerular filtration [5]. EFNB1 is involved in intestinal epithelial cell homeostasis [6]. We have shown that EFNB1 forward signalling through their Eph receptors can costimulate peripheral T cells in terms of enhancing cytokine production and proliferation *in vitro *and enhance thymocyte survival [7,8]. To further understand the role of EFNB1 in T cell development and function, we generated T-cell-specific gene knockout mice. The effects of EFNB1 on their immune system are presented here.

## Methods

### Generation of T cell-specific EFNB1 gene knockout mice

T cell-specific EFNB1 gene knockout mice were generated in our laboratory as described [9]. First, EFNB1 exon 1 was flanked with loxP sites. These floxed mice were backcrossed to the C57BL/6 background for more than nine generations and then mated with Lck-promoter-driven Cre transgenic (Tg) mice in the C57BL/6 background {strain B6.Cg-Tg(Lck-cre)I540Jxm/J, Jackson Laboratory, Bar Harbor, Maine, USA} to obtain T cell-specific EFNB1 gene knockout mice. Since EFNB1 is an X-linked gene, the deletion of an EFNB1 gene in one allele in males equals the deletion of the gene in two alleles in females. These mice were collectively called EFNB1^f/f/cre ^mice for convenience, regardless of gender. Floxed males or females without the Cre transgene were used as controls and were collectively called EFNB1^f/f ^mice.

All mice were housed under specific pathogen-free conditions and used in accordance with a protocol approved by the Institutional Animal Protection Committee of the University of Montreal Hospital Center (NO9055JWs).

### Immunofluorescent microscopy

One hundred thousand thymocytes in single cell suspension were applied to glass slides using Statspin^® ^cytofuge (IRIS international INC., Westwood, MA, USA) at 850 RPM for 4 min. Cells were fixed with 4% paraformaldehyde for 15 min and then blocked with PBS containing 10% FBS. Cells were then stained with 4 μg/ml goat anti-EFNB1 Ab (Clone 94038, R&D systems, Minneapolis, MN, USA) overnight at 4°C, followed by 0.25 μg/ml PE-donkey anti-goat IgG Ab (Jackson ImmunoResearch Laboratories, Inc., West Grove, PA, USA) for 45 min. The slides were mounted with prolong^® ^Gold antifade reagent (Invitrogen, Eugene, OR) and examined under a Carl Zeiss microscope using AxioVision™ software (Jena, Germany).

### Flow Cytometry

Single cell suspensions from the thymus and spleen were prepared and stained for flow cytometry as described in our previous publications (Yu et al., 2004; Yu et al., 2006). For intracellular antigen staining, cells were first stained with Abs against cell surface antigens fixed with Cytofix/Cytoperm solution (BD Biosciences, San Diego, CA, USA), then stained with Ab against intracellular antigens. Next, Abs were used for flow cytometry as follows: anti-mouse CD4 PerCP (0.2 μg/μl, Clone no. RM4-5); anti-mouse CD8 APC (0.2 μg/μl, Clone no. 53-6.7); anti-mouse CD25 APC (0.2 μg/μl, Clone no. 7D4); anti-mouse CD69 FITC (0.2 μg/μl, Clone no. H1.2F3); anti-mouse Thy1.2 PE (0.2 μg/μl, Clone no. 53-2.1); anti-mouse B220 APC-Cy™ 7 (0.2 μg/μl, Clone no. RA3-6B2); anti-mouse CD3 APC (clone no. 2C11); anti-mouse CD62L PE (clone no. MEL-14); anti-mouse CD45.1 FITC (clone no.A20); anti-mouse CD45.2 PerCP (clone no.104); anti-mouse IFN-γ FITC (0.2 μg/μl, Clone no. B27); anti-mouse IL4 PE (0.2 μg/μl, Clone no. BVD4-1D11); anti-mouse IL17 PE (0.2 μg/μl, Clone no. TC11-18H10) (BD Bioscience Pharmingen, San Diego, CA, USA); anti-mouse CD44 Pacific bue (clone no. 1M7, BioLegend, San Diego, CA); and anti-mouse FoxP3 APC (0.2 μg/μl, Clone no. FJK-16s) (eBioscience, San Diego, CA, USA).

### T cell activation and proliferation assay

Total spleen cells were stimulated with soluble hamster anti-mouse CD3 mAb (100 ng/ml, clone 2C11) plus rat anti-mouse CD28 mAb (1 μg/ml, clone no. 37.51.1). After 16 h culture, cells were stained with PE-rat anti-mouse Thy1.2 (clone no.53-21) and FITC-rat anti-CD25 (clone no.7D4) or anti-CD69 mAb (clone H1.2F3), and analyzed with 2-color flow cytometry. For T cell proliferation, spleen T cells were purified using Easysep^® ^Mouse T cell enrichment kit (Stemcell technologies, Vancouver, BC, Canada) and cultured in wells coated with hamster anti-mouse CD3 mAb (1 μg/ml for coating) alone or hamster anti-mouse CD3 mAb (0.1 μg/ml for coating) plus rat anti-mouse CD28 (1 μg/ml for coating). ^3^H-thymidine uptake of the cultured cells was measured at 24 h, 48 h and 72 h after culture, as described in our previous publication [7].

### Generation of bone marrow chimeras

Eight to 10-week-old C57BL/6 (CD45.2^+^) × C57/B6.SJL(CD45.1^+^) F1 mice were irradiated at 1,100 Rads. Twenty-four hours later, they received 4x10^6 ^T cell-depleted bone marrow cells from. Efnb1^f//f/cre ^alone or C57/B6.SJL and Efnb1^f//f/cre ^mice in a 1:1 ratio. Efnb1^f//f ^mouse bone marrow was used as a control. Spleen cells of the recipients were analyzed by flow cytometry 8 to 10 weeks following the bone marrow transplantation (BMTx).

### *In vitro *Th1, Th2, Th17 and Treg polarization

Th1, Th2, Th17 and Treg populations were polarized from naïve CD4^+ ^T cells that were isolated from pooled splenocytes and lymph node cells using Naïve CD4^+ ^T Cells Isolation Kits (R&D Systems). Purity of the naïve CD4^+ ^cells was routinely greater than 95%. Purified naïve T cells (0.1 × 10^6^/well) were mixed with T cell-depleted irradiated (3000 Rads) C57BL/6 feeder splenocytes (0.5 × 10^6 ^cells/well), and cultured in 96-well plates in RPMI medium 1640 containing 10% FCS, 100 μg/ml streptomycin, 100 units/ml penicillin G, 1× nonessential amino acids, 1 μM sodium pyruvate, 2.5 μM β-mercaptoethanol, and 2 μg/ml soluble hamster anti-CD3ε mAb (clone 145-2C11; 2 μg/ml). For Th1 polarization, rmIL-12 (10 ng/ml) and rat anti-IL-4 mAb (10 μg/ml, clone 11B11) were added to the culture. For Th2 polarization, rmIL-4 (20 ng/ml), rat anti-IL-12 mAb (Clone no.48110 10 μg/ml,) and rat anti-IFN-γ mAb (10 μg/ml, Clone no 37895) were added. For Th17 polarization, cultures were supplemented with recombinant mouse IL-6 (20 ng/ml), recombinant human TGF-β1 (5 ng/ml), and rat anti-IL-4 mAb and rat-anti-IFN-γmAb (10 μg/ml). For Treg polarization, recombinant human TGF-β1 (5 ng/ml) and rat anti-IL-4 and rat anti-IFN-γmAb (both at 10 μg/ml) were added to the culture. Recombinant cytokines and mAb against cytokines were all from R & D Systems (Minneapolis, MN, USA). Five days after culture, 5 nM of PMA, 500 ng/ml of ionomycin, and protein transport inhibitor BD GolgiStop™ (BD Bioscience; San Diego, CA, USA) were added for the last four hours of culture. Cells were harvested and stained for intracellular cytokines or FoxP3 followed by flow cytometry analysis.

## Results

### EFNB1 deletion in T cells

**Figure 1:**
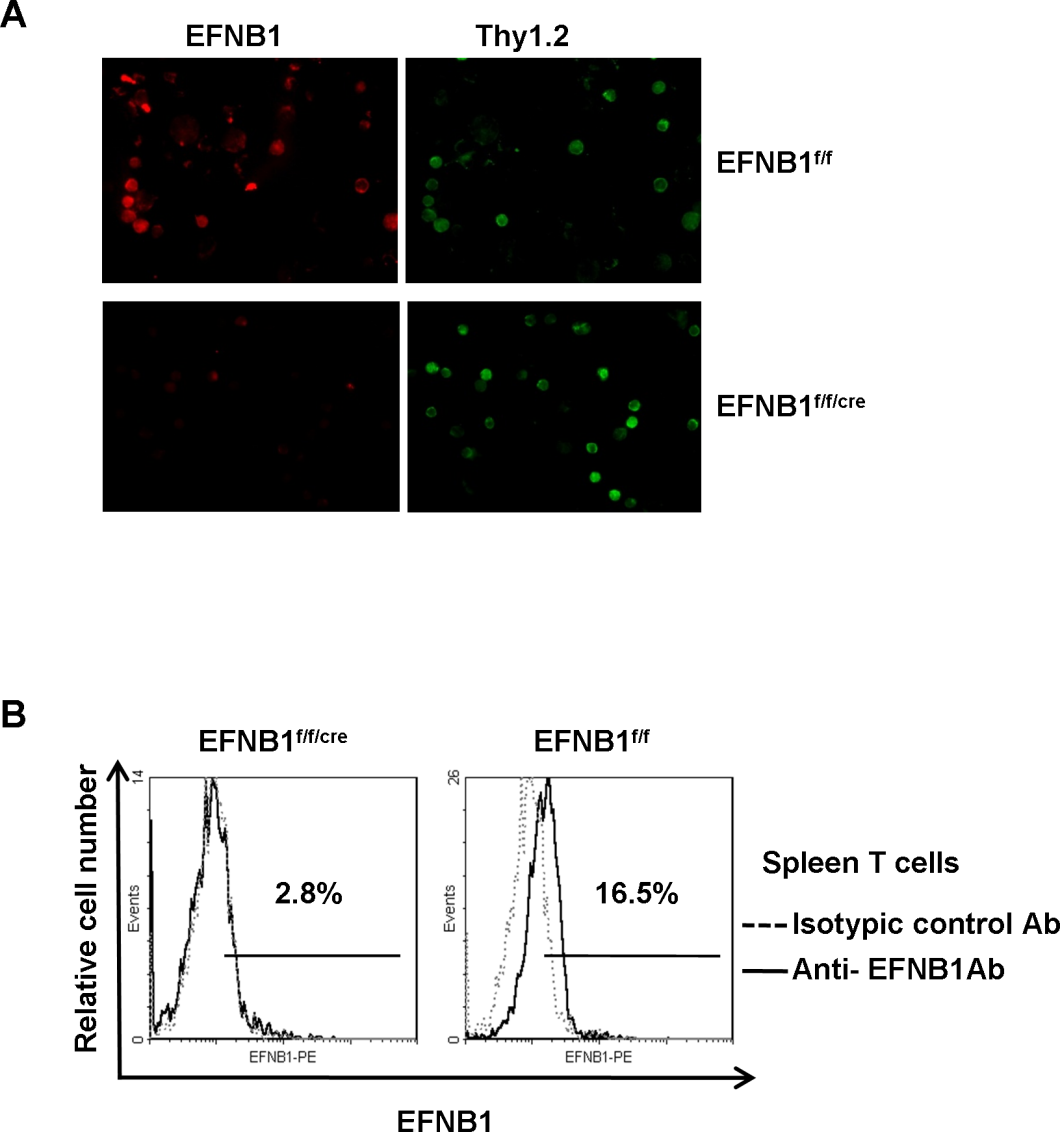
**T cell-specific deletion of EFNB1 in Lck-EFNB1^f/f ^mice according to immunofluorescent microscopy and flow cytometry**. *A*. Thymocytes from EFNB1^f/f ^(upper row) or Lck-EFNB1^f/f ^(bottom row) mice were stained with FITC-rat anti-Thy1.2 (green) and PE-rat anti-EFNB1 (red) mAbs. Micrographs of the cells with pseudocolouring are shown. *B*. CD3-gated spleen T cells from *EFNB1^f/f/cre ^*mice or *EFNB1^f/f ^*controls were analyzed for EFNB1 expression by flow cytometry. The dotted line represents isotypic control Ab, and solid line, anti-EFNB1 Abs. Percentage of EFNB1 positive cells was indicated. The experiments were repeated three times and data from representative ones are shown.

To confirm the deletion of EFNB1 in the T cell compartment, we conducted an immunofluorescence study on thymocytes using Thy1.2 and EFNB1 double staining. Control Thy1.2^+ ^thymocytes (in green) expressed EFNB1 (in red) (Figure 1A, upper row), while EFNB1 was not detectable in Thy1.2^+ ^thymocytes from EFNB1^f/f/cre ^mice (Figure 1A, bottom row). The deletion of EFNB1 was confirmed in mature spleen T cells by flow cytometry analysis (Figure 1B). These results demonstrated that EFNB1 was indeed deleted in the T-linage cells in KO mice.

### Cellularity and cell subpopulations in the thymus and spleen of *EFNB1^f/f/cre ^*mice

**Figure 2:**
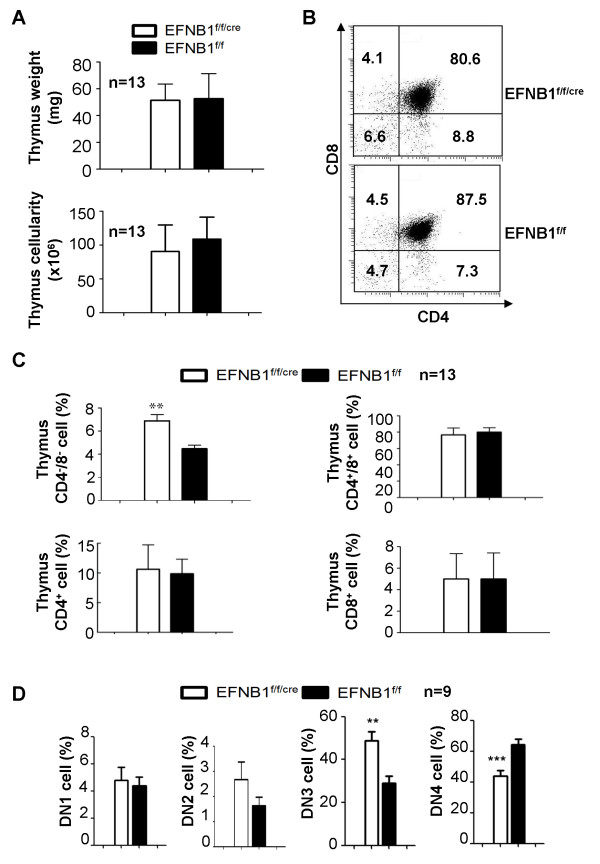
**Phenotype of Lck-EFNB1^f/f ^thymi**. *A. Thymus size and cellularity of Lck-EFNB1^f/f ^mice*. N = 13 pairs (*p *> 0.05; paired Student's t test). *B and C. Subpopulations of thymocytes from Lck-EFNB1^f/f ^and EFNB1^f/f ^mice according to flow cytometry*. Representative histograms are shown in B and bar graphs summarize data from 13 independent experiments are illustrated in C (*p *> 0.05; paired Student's *t *test). *D*. The percentages of DN1, DN2, DN3 and DN4 cells in Lck-EFNB1^f/f ^and EFNB1^f/f ^mice from 9 independent experiments are shown in bar graphs (paired Student's t test).

There was no gross morphological difference between EFNB1^f/f/cre ^and EFNB1^f/f ^thymi. They were of similar weight and cellularity, as shown in Figures 2. Thymocytes from EFNB1^f/f/cre ^mice were analyzed by flow cytometry for different cell populations. Representative histograms are shown in Figure 2B, and 2bar graphs in Figure 2C summarize the results of 13 independent experiments. EFNB1^f/f/cre ^and EFNB1^f/f ^mice had comparable percentages of CD4CD8 double positive (DP), CD4 single positive (SP), and CD8 SP thymocytes. Conversely, there was a moderate but significant increase in CD4CD8 double negative (DN) thymocytes in EFNB1^f/f/cre ^mice compared to EFNB1^f/f ^mice. Further analysis of DN thymocytes revealed that the augmented percentage of DN was due to the heightened percentage of DN3 stage cells in EFNB1^f/f/cre ^mice (Figure 2D).

**Figure 3:**
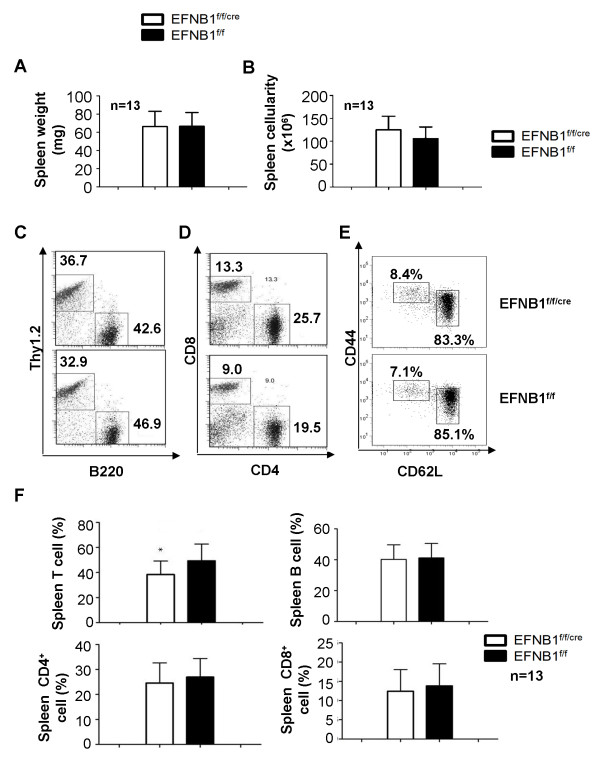
**Phenotype of Lck-EFNB1^f/f ^spleens**. *A and B. Spleen size and cellularity of Lck-EFNB1^f/f ^and EFNB1^f/f ^mice*. N = 13 pairs (*p*> 0.05; paired Student's t test). *C, D and E. Spleen T cell, B cell, CD4 T cell, CD8 T cell, CD4 naive cell and CD4 momery cell population of Lck-EFNB1^f/f ^mice according to flow cytometry*. Representative histograms are shown in C and D, and bar graphs summarize data from 13 independent experiments are illustrated in F (*p *> 0.05; paired Student's *t *test).

The spleen weight and cellularity of EFNB1^f/f/cre ^mice showed no significant difference compared to controls (Figures 3A &3B) after analyzing 13 pairs of mice. Different spleen cell populations were assessed by flow cytometry. Histograms from representative experiments are shown in Figures 3C and 3D, and a summary of results from 13 independent experiments are illustrated as bar graphs in Figure 3E. T and B cell populations in the EFNB1^f/f/cre ^spleens were comparable to those of EFNB1^f/f ^spleens (Figures 3C &3F). No apparent difference was found in the ratios of CD4 and CD8 cells between KO and control spleens (Figures 3D &3F). We next examined whether there is any change of naive versus memory population in EFNB1 KO T cell. As shown in 3E, no difference was observed in the percentage of CD44^lo^CD62L^hi ^EFNB1^f/f/cre ^naïve CD4 T cells and CD44^hi^CD62L^lo ^EFNB1 KO memory CD4 T cells compared with the littermate control CD4 T cells in the spleen.

**Figure 4:**
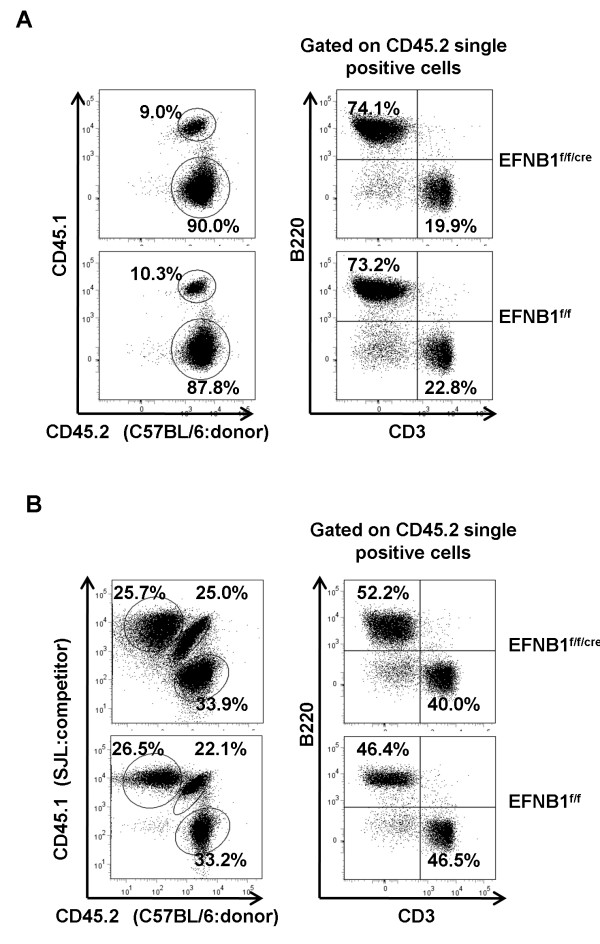
**Lck-EFNB1^f/f ^progenitors reconstitute the spleen in irradiated recipients**. *A*. 2 ×10^6 ^T-cell depleted bone marrow cells from Lck-Cre-EFNB1^f/f ^(top panel) or control EFNB1^f/f ^mice (bottom panel) were transferred to lethally irradiated C57BL/6 × SJL F1 recipients, followed by analysis 8 to 10 weeks later. Percentage of CD45.2^+ ^cells (derived from Lck-Cre-EFNB1^f/f ^or EFNB1^f/f ^bone marrow cells), and CD45.1^+^/CD45.2^+ ^cells (derived from residue cells of the recipients) is indicated. *B*. 1 × 10^6 ^T-cell depleted *Lck-Cre-EFNB1^f/f ^*and *EFNB1^f/f ^*bone marrow cells (CD45.2^+^) were mixed with T-cell depleted bone marrow cells from B6.SJL competitor at 1:1 ratio, and transplanted to lethally irradiated C57BL/6 × SJL F1 recipients. After 8 to 10 weeks, cells from spleen were analyzed for CD45.2 and CD45.1 staining. Percentage of CD45.2^+ ^cells (derived from Lck-Cre-EFNB1^f/f ^or EFNB1^f/f ^bone marrow cells), CD45.1^+ ^cells (derived from competing B6.SJL bone marrow cells), and CD45.1^+^/CD45.2^+ ^cells (derived from residue cells of the recipients) is indicated. In the WBI-BMTx models as described in Figure 4A and B, B220^+ ^B cells and CD3^+ ^T cells among CD45.2^+ ^cells (derived from Lck-Cre-EFNB1^f/f ^or EFNB1^f/f ^bone marrow cells) in the spleen were determined by flow cytometry, and their percentage is shown.

To determine the requirement for EFNB1 in early T cell development and T cell proliferation, we intraveneously transferred bone marrow hematopoietic progenitors from EFNB1^f/f/cre ^or EFNB1^f/f ^mice into lethally irradiated B6.SJL CD45.1 × C57BL/6 CD45.2 F1 hosts. In those single chimeras, the donor contribution (CD45.2 single positive) was about 90%. T-cell depleted bone marrow cells from EFNB1^f/f/cre ^or EFNB1^f/f ^generated similar sized pool of peripheral T cells (Figure 4A). We further examined T cell production of EFNB1^f/f/cre ^or EFNB1^f/f ^bone marrow progenitors in competitive repopulation experiment. To this end, we used a model of whole body irradiation (WBI) followed by bone marrow transplantation (BMTx), using B6.SJL bone marrow cells to compete with EFNB1^f/f/cre ^or EFNB1^f/f ^bone marrow cells. In this model, CD45.1 single positive cells were derived from competitor B6.SJL bone marrow cells; CD45.2 single positive cells were derived from EFNB1^f/f/cre ^or EFNB1^f/f ^bone marrow cells; and CD45.1/CD45.2 double positive cells were derived from residual recipient bone marrow cells and peripheral cells. As shown in Figure 4B, there was similar amount of the spleen cells derived from donor bone marrow cells (CD45.2 single positive cells) and competitor B6.SJL bone marrow cells (CD45.1 single positive cells) no matter whether the donor bone marrow cells derived from EFNB1^f/f/cre ^or EFNB1^f/f ^mice. While gated on CD45.2 single positive cells, no significant differences were found in the repopulated spleen T cells of the recipients received bone marrow cells from mixture of EFNB1^f/f/cre ^and B6.SJL mice compared with that of the recipients received bone marrow cells from mixture of control EFNB1^f/f ^and B6.SJL mice. This indicates that the EFNB1^f/f/cre ^bone marrow cells were comparable to the EFNB1^f/f ^control bone marrow cells in their capacity to compete with B6.SJL bone marrow cells to develop and expand in the void niche created by irradiation. Taken together, the results show that T cell development was minimally affected in the absence of EFNB1.

### *In vitro *activation and proliferation of EFNB1^f/f/cre ^T cells were not compromised

**Figure 5:**
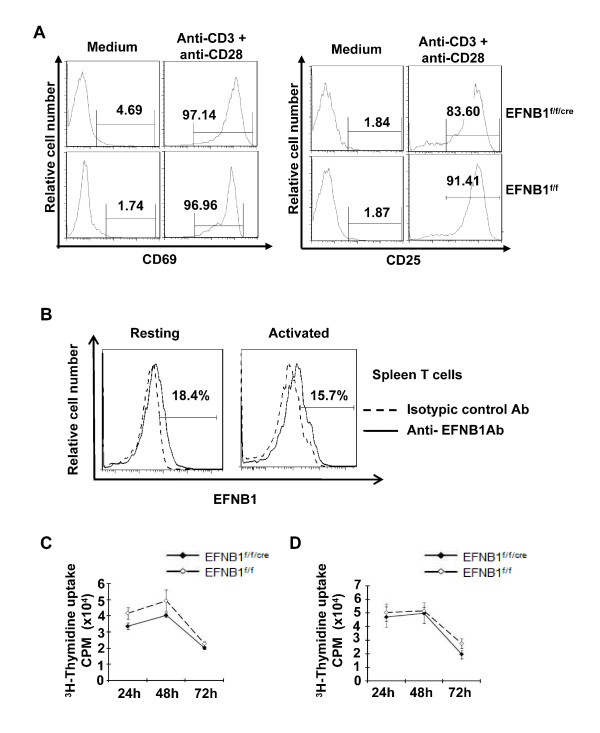
**Normal activation and proliferation of Lck-EFNB1^f/f ^T cells**. *A. Activation marker expression*. Total spleen cells from *Lck-EFNB1^f/f ^and EFNB1^f/f ^*mice were stimulated with soluble anti-CD3 mAb and anti-CD28 mAbs for 48 hours, and stained with PE-rat anti-mouse Thy1.2 and FITC-rat anti-mouse CD25 or CD69, followed by flow cytometry analysis. *B. EFNB1 expression in activated T cells*. Total spleen cells from EFNB1 f/f mice were cultured in medium or with anti-CD3 mAb for 48 hours, and stained with Thy1.2 -FITC and PE-rat anti-EFNB1 mAbs. The percentage of EFNB1 positive cells gated on T cells is indicated. *C and D. T cell proliferation*. Purified T cells from *Lck-EFNB1^f/f ^and EFNB1^f/f ^*mice were cultured in wells coated with solid phase anti-CD3 (C) or anti-CD3 plus anti-CD28 mAb (D) for 24 to 72 h. The cells were pulsed with^3 ^H-thymidine for the last 16 h of the culture. Samples were in triplicate. Mean ± SD of cpm are shown. Experiments were repeated more than 5 times and representative data are shown.

Next, we investigated the function of peripheral EFNB1^f/f/cre ^T cells in terms of activation and proliferation. Spleen T cells were purified by negative selection using magnetic beads; the purified cells contained more than 95% CD3 positive cells. They were cultured in wells coated with anti-CD3 and anti-CD28. After overnight culture, they were analyzed for the expression of activation markers such as CD25 and CD69. More than 95% of the T cells from EFNB1^f/f/cre ^mice upregulated their CD25 and CD69 expression within 16 h; such upregulation was comparable to that of T cells from EFNB1^f/f ^mice (Figure 5A). Meanwhile, no significant change of EFNB1 expression was noted between resting and activated T cells from EFNB1^f/f ^mice (Figure 5B).

We then examined proliferation of T cells from EFNB1^f/f/cre ^mice. Purified spleen T cells were cultured in wells coated with anti-CD3 mAb (1 μg/ml for coating), or anti-CD3 plus anti-CD28 mAb (0.1 μg/ml and 1 μg/ml for coating, respectively); and their proliferation was determined at 24, 48 and 72 h according to^3^H-thymidine uptake. A summary of three independent experiments is illustrated in Figures 4B and 4C. Overall, EFNB1^f/f/cre ^T cells showed no compromise in their ability to proliferate upon solid phase anti-CD3 mAb (Figure 5C) or anti-CD3 plus anti-CD28 mAb (Figure 5D) stimulation. Of importance, anti-CD3 Ab concentration (1 μg/ml) was higher in wells coated with anti-CD3 Ab alone, compared to the concentration used in wells coated with anti-CD3 plus anti-CD28 Abs. This shows that the maximal proliferation under these two conditions was similar.

### *In vitro *differentiation of EFNB1^f/f/Cre ^T cells

**Figure 6:**
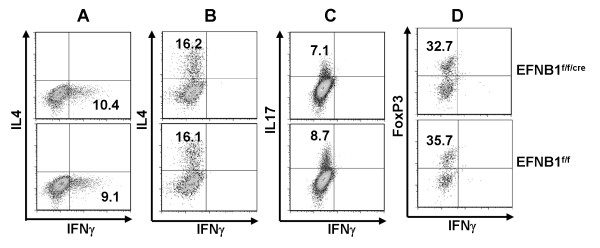
**CD4 cell in vitro differentiation**. Naïve CD4 cells were cultured under conditions favouring Th1 (A), Th2 (B), Th17 (C) and Treg (D) cells. Their intracellular cytokine or FoxP3 expression was determined by flow cytometry. Experiments were repeated more than 5 times and representative data are shown.

Since EFNB1^f/f/Cre ^T cells showed no abnormality in activation and proliferation, we next assessed whether they could properly differentiate into different functional subpopulations. Spleen naive CD4 T cells from EFNB1^f/f/Cre ^and control EFNB1^f/f ^mice were cultured under conditions favouring the development of Th1 (Figure 6A), Th2 (Figure 6B), Th17 (Figure 6C) and Treg (Figure 6D) cells. After optimal durations of culture for each type of cell, the cells were collected and analyzed for their intracellular IFN-γ, IL4, IL17 and FoxP3 content by flow cytometry. After differentiation, as shown in Figures 6A-6D, EFNB1^f/f/Cre ^and EFNB1^f/f ^CD4 cells were comparable in their percentage of intracellular IFN-γ^+^, IL4^+^, IL17^+ ^and FoxP3^+ ^cells. This indicates that EFNB1^f/f/Cre ^CD4 cells are capable of normal differentiation into Th1, Th2, Th17 and Treg cells.

## Discussion

We investigated T cell development in mice with T cell-specific deletion of EFNB1. Results show that in the absence of EFNB1, the thymus and spleen showed mostly normal subpopulations of T cell origin. In single chimeras and competitive chimaeras, the EFNB1^f/f/cre ^bone marrow cells demonstrated comparable T cell development as the control EFNB1^f/f ^bone marrow cells. Moreover, mature T cells had no apparent defects in their activation and proliferation or in their ability to differentiate into functional Th1, Th2, Th17 and Treg cells.

Previous *in vitro *studies using foetal thymus organ culture have shown that recombinant EFNB1 protein is capable of influencing T cell development and enhancing thymocyte survival [8]. In another study [7], solid phase EFNB1 was shown to increase mature T cell response to TCR stimulation. How do we reconcile these observations with the present findings that EFNB1 deletion showed no apparent impact on T cell development and mature T cell function? As mentioned in the introduction, EFNB1 promiscuously interacts with multiple EphB family members such as EphB1, B2, B3, B4, and B6, as well as with a few EphA family members such as EphA4 and EphA3. When soluble EFNB1 is introduced into thymus organ culture, it not only blocks the interaction of multiple Ephs with EFNB1, but also likely interferes with interactions between those Ephs and other EFN members. When EFNB1 is placed on solid phase, it will noticeably trigger signalling of multiple Ephs. Such effects cannot be achieved by simple EFNB1 deletion. In the absence of EFNB1, its forward signalling to other Eph molecules and its reverse signalling are likely compensated by other EFNs.

The absence of a demonstrated phenotype in T cell development and function following EFNB1 deletion does not prove that this molecule is unimportant. On the contrary, this observation suggests an essential importance of EFNB1. It can be argued that the role of EFNB1 in the T cell compartment is so essential, that any accidental mutation could lead to disastrous consequences. As a counter measure to prevent such disasters from occurring, through evolution, we have developed highly redundant Eph and EFN systems, as well as promiscuous interactions between Eph and EFN members. With such a system, any aberrant mutation that occurs to certain Eph or EFN members will be safely compensated by others in such a way as to guarantee their normal function in the T cell compartment. It is further inferred that the true importance of EFNs can only be revealed if a deletion of multiple EFN family members occurs at the same time. Through our studies on T cell-specific deletion of both EFNB1 and EFNB2 in mice [9]), we found that in the absence of both EFNB1 and EFNB2, there was compromised αβT cell development in the thymus along with abnormal thymic structure. The EFNB1/EFNB2 KO T cells significantly failed to compete with normal T cells during homeostatic expansion in irradiated recipients. EFNB1/EFNB2 double KO T cells were significantly inferior to normal T cells in their ability to differentiate into Th1 and Th17 cells. Such a handicap resulted in compromised *in vivo *T cell-mediated immune responses such as allograft rejection and anti-virus immunity. These results confirm the critical role of EFNB1 in T cell development and function. However, this role was not observed in the EFNB1 single KO mice used in this study.

## Conclusions

To conclude, we are the first to have carried out an analysis of roles of T cell development and function in the mice with conditional EFNB1 deletion in the T cell compartment, though the effect of *the single EFNB1 KO *appears to be minimal. Our results suggest that the function of EFNB1 in the T cell compartment could be compensated by other members of the EFN family, and that such redundancy safeguards the pivotal roles of EFNB in T cell immunity. Additional studies on EFNB family, i.e. EFNB1, EFNB2 and EFNB3, double or even triple null mutated mice to investigate T cell development and function are warranted to confirm such indications.

## Abbreviations

CRCHUM: Centre de recherche de Centre hospitalier de l'Université de Montréal; DN: double negative; DP: double positive; EFNs: ephrins; KO: knockout; Tg: transgenic; SP: single positive

## Authors' contributions

All authors participated in experimental procedures leading to figures contained within. WJ and HL participated in writing of the manuscript. All authors have read and approved the final version of the manuscript.
